# Association of type 2 diabetes mellitus and antidiabetic medication with risk of prostate cancer: a population-based case-control study

**DOI:** 10.1186/s12885-020-07036-4

**Published:** 2020-06-15

**Authors:** E. Lin, Hans Garmo, Mieke Van Hemelrijck, Jan Adolfsson, Pär Stattin, Björn Zethelius, Danielle Crawley

**Affiliations:** 1 School of Cancer and Pharmaceutical Sciences, Translational Oncology and Urology Research (TOUR), King’s College London, London, UK; 2 Department of Clinical Science, Intervention and Technology, Karolinska Institute, Stockholm, Sweden; 3 Department of Surgical Sciences, Uppsala University, Uppsala, Sweden; 4 Department of Public Health/Geriatrics, Uppsala University, Uppsala, Sweden

**Keywords:** Type 2 diabetes mellitus, Antidiabetic medication, Duration, Prostate cancer risk

## Abstract

**Background:**

Prostate cancer (PCa) and type 2 diabetes mellitus (T2DM) are prevalent conditions that often occur concomitantly. However, many aspects of the impact of T2DM, particularly the duration of T2DM and antidiabetic medications, on PCa risk are poorly understood.

**Methods:**

To assess the association of duration of T2DM and antidiabetic medication with PCa risk, we designed a matched case-control study, including 31,415 men with PCa and 154,812 PCa-free men in Prostate Cancer data Base Sweden (PCBaSe) 4.1.

**Results:**

Overall, a decreased risk of PCa was observed for men with T2DM (odds ratio (OR): 0.81, 95% confidence interval (CI): 0.78–0.84), as compared to men without T2DM. The decreased risk of PCa was consistently showed across duration of T2DM. With respect to use of antidiabetic drugs, this inverse association with duration was also found for all medications types, as compared to men without T2DM, including insulin, metformin and sulphonylurea (SU) (e.g. 3- < 5 yr insulin OR:0.69, 95%CI:0.60–0.80; 3- < 5 yr metformin OR: 0.82, 95%CI: 0.74–0.91; 3- < 5 yr SU OR: 0.72, 95%CI: 0.62–0.83). When stratifying by PCa risk categories, this decreased risk was most evident for diagnosis of low and intermediate-risk PCa (low-risk OR: 0.65, 95%CI: 0.66–0.70, intermediate-risk OR: 0.80, 95%CI: 0.75–0.85).

**Conclusions:**

The study showed an inverse association between pre-existing T2DM and PCa across different durations of T2DM and all types of T2DM medication received. This inverse association was most evident for low- and intermediate-risk PCa, suggesting that whilst T2DM and its medication may protect some men from developing PCa, the relationship warrants further study.

## Background

Prostate cancer (PCa) and type 2 Diabetes Mellitus (T2DM) are prevalent conditions that often occur concomitantly [1]. Pre-existing T2DM has been associated with a decreased risk of PCa in multiple observational studies [2–8]. Existing studies examining the association of antidiabetic medications with PCa risk have, however, produced inconsistent results. While some suggest an inverse association of antidiabetic medications, particularly metformin, with PCa risk [9–13], others suggest null associations [14–17]. These existing studies have not explored the effect of the duration of antidiabetic medications on PCa risk. Moreover, few studies have assessed the impact of antidiabetic medications on severity of PCa at the time of diagnosis, i.e. PCa risk categories.

In a previous study using Prostate Cancer data Base Sweden (PCBaSe) and the National Diabetes Register data, Fall et al. (2013) reported reduced risk of PCa across all PCa risk categories (OR = 0.80, 95%CI = 0.76–0.86) for men with T2DM compared to men without T2DM, especially low-risk PCa (OR = 0.71, 95%CI = 0.64–0.80) [4]. Since 2013, the National Diabetes Register have improved completeness of their data. In addition, PCBaSe has now been linked to the National Prescribed Drug Register to assess what medications patients receive for T2DM. Here, we use updated PCBaSe data to examine the association between duration of T2DM and antidiabetic medications and PCa risk.

## Methods

### Participants

We conducted a case-control study using PCBaSe 4.1 which is based on the National Prostate Cancer Register (NPCR) of Sweden, a nationwide population-based register starting in 1998 that includes more than 98% of the men registered with PCa in the National Cancer Register. NPCR contains detailed data on cancer characteristics including prostate-specific antigen (PSA) at the time of diagnosis, staging and Gleason Grade Groups [18]. Using the unique personal identity number given to all residents in Sweden, all participants have been subsequently linked to Swedish health care registers and demographic databases. These include the National Patient Register and Longitudinal integrated database for health insurance and labour market studies, to obtain data on co-morbidities, civil status and educational level in 2008 [18]. Comorbidities were quantified using the Charlson Comorbidity Index (CCI), based on discharge diagnoses in the In-Patient Registry with different weights (1, 2, 3 and 6) to each ICD code [19, 20]. For the current study, diabetes was not included in the CCI.

For every case in PCBaSe, five PCa-free control men (*n* = 154,812) were randomly selected from the general Swedish population by use of the personal identity number [21], matched on year of birth and county of residence. The National Diabetes Register was initiated in 1996, and data records were linked via the personal identity number to PCBaSe [1]. In 2013, approximately 92% of the prevalent cases of type 1 and type 2 diabetes mellitus were included in the National Diabetes Register. We also used data from the National Prescribed Drug Register. The National Prescribed Drug Register was established in July 2005 and contains all prescribed drugs dispensed at pharmacies in Sweden.

### Identification of men with T2DM

To investigate the association between T2DM and PCa, we assessed data related to T2DM diagnosis and drug treatments available in the National Diabetes Register and National Prescribed Drug Register. We defined pre-existing T2DM as fulfilling at least one of following criteria:

1. a medical record of the year of T2DM onset in the National Diabetes Register
2. a registration date of T2DM in the National Diabetes Register
3. a prescription for antidiabetic drugs (at least two filled prescriptions records) in the National Prescribed Drug Register.

### Exposure

The primary exposure was duration of T2DM. The duration of T2DM was calculated using the year of T2DM onset or the registration date of T2DM in the National Diabetes Register (94%), or the duration of antidiabetic medications in the National Prescribed Drug Register (6%).

In addition, we also used duration of antidiabetic medications, including insulin, metformin, and sulphonylurea (SU) as documented in the National Prescribed Drug Register up to 8.5 years prior to the date of PCa diagnosis (July 2005 – December 2016). As the Swedish drug reimbursement system is based on records of patients’ visiting to the pharmacies every 3 months, the 8.5-year period in the National Prescribe Drug Register was divided into 34 sub-periods, which were shown as 34 filled prescription records in the dataset of the study. Each filled prescription record equates to 3 months’ antidiabetic medication. Men with at least two filled prescription records of the same antidiabetic medication were assumed to be taking that antidiabetic drug as a treatment of T2DM. The duration of an antidiabetic drug was defined by the number of the corresponding records found in the National Prescribed Drug Register.

Use of metformin was divided into high-dose and low-dose groups with a cut-off at 1 g of metformin per day. We categorized duration into 3 groups: (i) ≥0.5 year - < 3 years, (ii) ≥3 year - < 5 years, and (iii) 5 year - ≤8.5 years. We calculated duration of insulin, metformin, and SU separately, even if some men received more than one antidiabetic drug. For example, if a man took high-dose metformin and insulin at the same time, he contributed time to the duration of both high-dose metformin and insulin.

### Outcome

The primary outcome of the study was diagnosis of PCa, and we also obtained information on PCa risk categories from the NPCR. According to the modified version of the National Comprehensive Cancer Network (NCCN) guideline, men with PCa can be categorized into five risk categories: i) low-risk: T1 or T2a stage, PSA < 10 ng/mL and Gleason score 6; ii) intermediate-risk: T2b or T2c stage, 10 ng/mL, PSA < 20 ng/mL, or Gleason score 7; iii) high-risk: T3a or T4 stage, PSA ≥ 20 ng/mL, or Gleason score ≥ 8; iv) regional metastases any T, N1 and M0 stage; v) distant metastases, any T or N and M1 stage [22]. In the main analysis, we included men with high-risk, regional metastases, and distant metastases into the high-risk category group to increase the power. We also conducted a subgroup analysis for each category.

### Statistical analysis

Conditional logistic regression was used to calculate odds ratios (OR) and 95% confidence intervals (CI) for PCa risk. Models were adjusted for education levels (high, middle and low), civil status (married, not married, widowed and divorced), CCI and age at the year of PCa diagnosis.

All data management was performed with STATA 15.1. The study has been approved by The Research Ethics Board at Uppsala University, Sweden.

## Results

31,415 men with PCa and 154,812 PCa-free men were included in the study. Table 1 shows their baseline characteristics. Of the men with PCa, 4186 (13%) had T2DM, whereas among the controls 24,965 (16%) had T2DM. Age, education level, civil status and CCI were similar for cases and controls (Table 1). PCa men with T2DM were more likely to be diagnosed with advanced PCa or metastatic disease and had higher PSA and Gleason grade group 4 & 5 compared with PCa men without T2DM, as shown in Table 2.

**Table 1:** Characteristics of men in PCBaSe 4.1 diagnosed with prostate cancer between 2014 and 2016 (*n* = 31,415) and their matched controls (*n* = 154,812)

	Controls, ***N*** = 154,812		Cases, ***N*** = 31,415	
	***N***	%	***N***	%
**Age (year),*n*(%)**				
< 50	1617	1.0	326	1.0
50–59	17,806	11.5	3625	11.5
60–69	61,287	39.6	12,514	39.8
70–79	54,062	34.9	10,900	34.7
80+	20,040	12.9	4050	12.9
**Education level,*n*(%)**				
Low	48,882	31.6	9311	29.6
Middle	79,904	51.6	16,553	52.7
High	24,159	15.6	5348	17.0
Missing	18,67	1.2	203	0.6
**Civil status,*n*(%)**				
Married	92,576	59.8	20,122	64.1
Not married	24,431	15.8	4120	13.1
Divorced	26,647	17.2	4946	15.7
Widower	11,151	7.2	2216	7.1
Missing	7	0.0	11	0.0
**CCI,*n*(%)**				
0	113,602	73.4	23,616	75.2
1	20,102	13.0	3734	11.9
2	12,032	7.8	2501	8.0
3+	9076	5.9	1562	5.0

Numbers may not add up to 100% due to rounding

*Abbreviations*: *N* number, *CCI* Charlson Comorbidity Index

**Table 2:** Characteristics of men in PCBaSe 4.1 diagnosed with prostate cancer between 2014 by T2DM status

	No T2DM^**1**^, ***N*** = 27,229		T2DM, ***N*** = 4186	
	***N***	%	***N***	%
**T-stage,*n*(%)**				
T1a	642	2.4	146	3.5
T1b	331	1.2	90	2.2
T1c	13,288	48.8	1640	39.2
T2	7999	29.4	1328	31.7
T3	3593	13.2	713	17.0
T4	617	2.3	120	2.9
T0, Tx, Missing	759	2.8	149	3.6
**N-stage,*n*(%)**				
N0	9112	33.5	1355	32.4
N1	1346	4.9	223	5.3
Nx, Missing	16,771	61.6	2608	62.3
**M-stage,*n*(%)**				
M0	24,781	91.0	3690	88.2
M1	2423	8.9	493	11.8
Missing	25	0.1	3	0.1
**PSA,*n*(%)**				
PSA < 4	3526	12.9	388	9.3
4 ≤ PSA < 10	12,373	45.4	1613	38.5
10 ≤ PSA < 20	4750	17.4	874	20.9
20 ≤ PSA < 50	2945	10.8	555	13.3
50 ≤ PSA < 100	1143	4.2	263	6.3
PSA ≥ 100	2492	9.2	493	11.8
**Gleason Grade Group**^**2**^**,*n*(%)**				
Grade group 1	10,152	37.3	1152	27.5
Grade group 2	7183	26.4	1056	25.2
Grade group 3	3496	12.8	645	15.4
Grade group 4	2397	8.8	470	11.2
Grade group 5	3253	11.9	715	17.1
Missing	748	2.7	148	3.5

Numbers may not add up to 100% due to rounding

^2^ Gleason grade group 1 = Gleason Score ≤ 6, Grade group 2 = Gleason Score 3 + 4 = 7, Grade group 3 = Gleason Score 4 + 3 = 7, Grade group 4 = Gleason Score 4 + 4, 3 + 5, 5 + 3 = 8, Grade group 5 = Gleason Score 9 and 10

*Abbreviations*: *N* number, *T2DM* type 2 diabetes mellitus, *PSA* Prostate-Specific Antigen

### The association of duration of T2DM and antidiabetic medications with PCa risk

Overall, a decreased risk of PCa was observed for men with T2DM (OR: 0.81, 95% CI: 0.78–0.84), as compared to men without T2DM.

There was a consistent reduction of PCa risk over all durations of T2DM, except for men who were diagnosed of T2DM less than 1 year prior to PCa diagnosis. We found a 19% reduction in risk of PCa among men with T2DM for ≥1 year and < 5 years and a 27% reduction in risk of PCa for men with pre-existing T2DM for at least 20 years as compared to men without T2DM (Table 3). Men on insulin, high-and low-dose metformin, and SU showed a persistently decreased risk of PCa over all durations (Table 3).

**Table 3:** Odds ratios and 95% CIs for risk of prostate cancer according to type 2 diabetes mellitus (T2DM) status and T2DM medications

Dependent variables	Controls	Cases	Model 1^**1**^		Model 2^**2**^	
	***N*** = 154,812	***N*** = 31,415	OR	95% CI	OR	95% CI
**T2DM**						
no DM	129,847	27,229	Ref		Ref	
T2DM	24,965	4186	0.80	(0.77–0.83)	0.81	(0.78–0.84)
**Duration of T2DM (years)**						
no DM	129,847	27,229	Ref		Ref	
< 1	1306	307	1.12	(0.99–1.27)	1.15	(1.01–1.30)
1 - < 5	5983	990	0.79	(0.74–0.84)	0.81	(0.75–0.86)
5 - < 10	6862	1144	0.79	(0.74–0.85)	0.81	(0.76–0.86)
10 - < 20	7724	1269	0.78	(0.73–0.83)	0.79	(0.75–0.84)
20	3090	476	0.73	(0.66–0.81)	0.73	(0.67–0.81)
**Duration of Insulin (years)**						
no DM	129,847	27,229	Ref		Ref	
0.5 - < 3	2370	335	0.67	(0.59–0.75)	0.68	(0.60–0.76)
3 - < 5	1498	208	0.67	(0.58–0.77)	0.69	(0.60–0.80)
5	4323	645	0.71	(0.65–0.77)	0.72	(0.66–0.79)
Missing	16,774	2998				
**Duration of high-dose Metformin (years)**						
no DM	129,847	27,229	Ref		Ref	
0.5 - < 3	6339	1010	0.76	(0.71–0.82)	0.77	(0.72–0.83)
3 - < 5	2450	410	0.80	(0.72–0.90)	0.82	(0.74–0.91)
5	2117	376	0.86	(0.77–0.96)	0.87	(0.78–0.98)
Missing	14,059	2390				
**Duration of low-dose Metformin (years)**						
no DM	129,847	27,229	Ref		Ref	
0.5 - < 3	10,069	1588	0.75	(0.71–0.80)	0.77	(0.72–0.81)
3 - < 5	3926	663	0.80	(0.74–0.87)	0.82	(0.75–0.89)
5	1918	343	0.85	(0.76–0.96)	0.87	(0.77–0.97)
Missing	9052	1592				
**Duration of SU (years)**						
no DM	129,847	27,229	Ref		Ref	
0.5 - < 3	2604	369	0.68	(0.61–0.76)	0.68	(0.61–0.76)
3 - < 5	1402	216	0.72	(0.62–0.83)	0.72	(0.62–0.83)
5	1903	293	0.74	(0.66–0.84)	0.76	(0.67–0.86)
Missing	19,056	3308				

^1^ Crude; ^2^ adjusted for CCI, education level, civil status and the age at year of PCa onset

*Abbreviations*: *OR* odds ratio, *95% CI* 95% confidence interval, *PCa* prostate cancer, *T2DM* type 2 diabetes mellitus, *SU* sulfonylurea

### The association of duration of T2DM and antidiabetic medications with PCa risk categories

There was a decreased risk of low- and intermediate-risk PCa for men with T2DM, as compared to men without T2DM (Table 4). The decreased risk persisted over time, with the largest reduction found for men whose duration of T2DM was 10–20 years in the low-risk category and over 20 years in intermediate-risk category. However, no association was found for the high-risk category.

**Table 4:** Association of T2DM medications with risk categories of prostate cancer 1

	Low-risk category				Intermediate-risk group				High-risk group 2			
	Controls (N)	Cases (***N***)	OR	95% CI	Controls (***N***)	Cases (***N***)	OR	95% CI	Controls (***N***)	Cases (***N***)	OR	95% CI
**T2DM**												
no DM	36,247	7781	Ref		43,691	9201	Ref		46,549	9563	Ref	
T2DM	5898	784	0.65	(0.66–0.70)	8280	1355	0.8	(0.75–0.85)	10,084	1910	0.92	(0.87–0.97)
**Duration of T2DM (years)**												
no DM	36,247	7781	Ref		43,691	9201	Ref		46,549	9563	Ref	
< 1	320	63	0.98	(0.75–1.29)	447	111	1.23	(0.99–1.51)	507	126	1.21	(0.99–1.47)
1 - < 5	1600	217	0.66	(0.57–0.76)	2021	331	0.81	(0.72–0.91)	2184	403	0.9	(0.81–1.01)
5 - < 10	1676	216	0.63	(0.54–0.72)	2292	379	0.8	(0.72–0.90)	2710	507	0.91	(0.82–1.00)
10 - < 20	1726	216	0.61	(0.53–0.71)	2520	401	0.78	(0.70–0.87)	3272	611	0.9	(0.83–0.99)
20	576	72	0.62	(0.48–0.79)	1000	133	0.65	(0.54–0.78)	1411	263	0.88	(0.77–1.01)
**Duration of Insulin (years)**												
no DM	36,247	7781	Ref		43,691	9201	Ref		46,549	9563	Ref	
0.5 - < 3	587	49	0.42	(0.31–0.56)	751	113	0.76	(0.62–0.94)	976	160	0.78	(0.65–0.92)
3 - < 5	369	37	0.53	(0.38–0.75)	506	57	0.57	(0.43–0.76)	574	101	0.86	(0.70–1.10)
5	908	98	0.55	(0.44–0.68)	1470	196	0.65	(0.56–0.76)	1829	339	0.89	(0.79–1.00)
Missing	4034	600			5553	989			6705	1310		
**Duration of high-dose Metformin (years)**												
no DM	36,247	7781	Ref		43,691	9201	Ref		46,549	9563	Ref	
0.5 - < 3	1609	207	0.63	(0.54–0.73)	2138	336	0.77	(0.68–0.86)	2418	435	0.87	(0.78–0.97)
3 - < 5	612	64	0.52	(0.40–0.68)	855	147	0.85	(0.71–1.01)	924	185	0.98	(0.83–1.15)
5	530	66	0.61	(0.47–0.79)	771	123	0.77	(0.64–0.94)	751	176	1.17	(0.99–1.38)
Missing	3147	447			4516	749			5991	1114		
**Duration of low-dose Metformin (years)**												
no DM	36,247	7781	Ref		43,691	9201	Ref		46,549	9563	Ref	
0.5 - < 3	2602	314	0.6	(0.53–0.68)	3470	513	0.73	(0.66–0.80)	3718	709	0.92	(0.85–1.00)
3 - < 5	920	119	0.63	(0.52–0.77)	1259	238	0.9	(0.78–1.04)	1634	287	0.86	(0.75–0.98)
5	401	56	0.67	(0.51–0.89)	607	103	0.84	(0.68–1.03)	844	173	1	(0.84–1.18)
Missing	1975	1297			2944	501			3888	741		
**Duration of SU (years)**												
no DM	36,247	7781	Ref		43,691	9201	Ref		46,549	9563	Ref	
0.5 - < 3	593	64	0.53	(0.41–0.69)	881	132	0.73	(0.61–0.89)	1059	170	0.76	(0.64–0.90)
3 - < 5	342	42	0.6	(0.43–0.83)	452	67	0.7	(0.54–0.91)	567	98	0.79	(0.64–0.99)
5	367	48	0.68	(0.50–0.92)	602	82	0.66	(0.52–0.84)	882	154	0.85	(0.72–1.02)
Missing	4596	630			6345	1074			7576	1488		

1 The results were adjusted for CCI, education level, civil status and the age at year of PCa onset

2 We combined high risk, reginal metastases, and distant metastases into high-risk category group

With respect to duration of antidiabetic medications, the reduction in PCa risk was again clearest for low and intermediate-risk PCa. For those taking insulin, there was a decreased risk of low-risk PCa (OR: 0.42, 95% CI: 0.31–0.56) at 0.5–3 years (Table 4). A similar association was seen for men taking high- and low-dose metformin and SU. For the intermediate-risk PCa category, there was a decreased risk with all durations of different antidiabetic medications. In contrast, no association was observed in high-risk PCa category (Table 4). No clear associations were observed inthe subgroup analysis,inwhich the high-risk category was further divided into: high-risk, regional metastases, and distant metastases (Table 5).

**Table 5:** Association of T2DM and antidiabetic medications with high-risk, regional metastases and distant metastases of prostate cancer 1

	High risk 2				Regional metastases				Distant metastases			
	Controls (***N***)	Cases (***N***)	OR	95% CI	Controls (***N***)	Cases (***N***)	OR	95% CI	Controls (***N***)	Cases (***N***)	OR	95% CI
**T2DM**												
no DM	24,746	5081	Ref		6772	1398	Ref		15,031	3084	Ref	
T2DM	5333	1011	0.93	(0.87–1.00)	1519	282	0.9	(0.78–1.04)	3232	617	0.91	(0.82–1.00)
**Duration of T2DM (years)**												
no DM	24,746	5081	Ref		6772	1398	Ref		15,031	3084	Ref	
< 1	257	67	1.28	(0.97–1.68)	92	15	0.76	(0.43–1.35)	158	44	1.33	(0.95–1.87)
1 - < 5	1122	224	0.99	(0.85–1.14)	350	62	0.87	(0.66–1.15)	712	117	0.8	(0.65–0.97)
5 - < 10	1453	281	0.95	(0.83–1.08)	396	68	0.84	(0.64–1.09)	816	158	0.87	(0.73–1.04)
10 - < 20	1727	316	0.9	(0.80–1.02)	467	94	0.97	(0.77–1.22)	1078	201	0.89	(0.76–1.04)
20	774	123	0.77	(0.63–0.94)	214	43	0.96	(0.69–1.35)	423	97	1.06	(0.84–1.32)
**Duration of Insulin (years)**												
no DM	24,746	5081	Ref		6772	1398	Ref		15,031	3084	Ref	
0.5 - < 3	509	80	0.77	(0.60–0.98)	150	16	0.52	(0.31–0.88)	317	64	0.9	(0.68–1.19)
3 - < 5	291	46	0.85	(0.63–1.16)	86	17	0.97	(0.57–1.65)	197	35	0.83	(0.58–1.20)
5	971	169	0.86	(0.72–1.01)	270	50	0.88	(0.64–1.21)	588	120	0.96	(0.78–1.18)
Missing	3562	713			1013	199			2130	398		
**Duration of high-dose Metformin (years)**												
no DM	24,746	5081	Ref		6772	1398	Ref		15,031	3084	Ref	
0.5 - < 3	1290	235	0.89	(0.77–1.03)	367	66	0.87	(0.66–1.15)	761	134	0.84	(0.69–1.02)
3 - < 5	508	97	0.96	(0.77–1.20)	125	33	1.23	(0.82–1.83)	291	55	0.89	(0.67–1.20)
5	408	95	1.19	(0.94–1.50)	112	23	0.96	(0.61–1.52)	231	58	1.25	(0.93–1.68)
Missing	3127	584			915	160			1949	370		
**Duration of low-dose Metformin (years)**												
no DM	24,746	5081	Ref		6772	1398	Ref		15,031	3084	Ref	
0.5 - < 3	1966	385	0.97	(0.86–1.09)	552	101	0.89	(0.71–1.12)	1200	223	0.86	(0.74–1.00)
3 - < 5	891	152	0.85	(0.72–1.02)	228	48	1	(0.73–1.38)	515	87	0.81	(0.64–1.02)
5	428	86	0.97	(0.76–1.23)	124	33	1.44	(0.97–2.15)	292	54	0.87	(0.65–1.17)
Missing	2048	388			615	100			1225	253		
**Duration of SU (years)**												
no DM	24,746	5081	Ref		6772	1398	Ref		15,031	3084	Ref	
0.5 - < 3	559	81	0.7	(0.55–0.88)	151	29	0.89	(0.58–1.36)	349	60	0.83	(0.62–1.09)
3 - < 5	302	54	0.85	(0.63–1.15)	78	15	0.95	(0.53–1.70)	187	29	0.65	(0.43–0.98)
5	469	69	0.74	(0.57–0.96)	128	22	0.81	(0.51–1.28)	285	63	1.07	(0.81–1.42)
Missing	4003	807			1162	216			2411	465		

1 The results were adjusted for CCI, education level, civil status and the age at year of PCa onset

2 Cases in “high risk” only refer to PCa men at T3a or T4 stage, whose PSA ≥ 20 ng/ml or Gleason Score ≥ 8, without regional metastases and distant metastases

## Discussion

In this nationwide, population-based case-control study, we observed that men with T2DM had a decreased risk of PCa for any duration of T2DM, as well as any exposure time to all types of antidiabetic medication. The reduction in risk was strongest for low- and intermediate- risk PCa, whereas there was no decrease for high-risk PCa.

### Duration of T2DM medication

We found a consistent reduction of PCa risk over all durations of T2DM, which supports previous studies [4, 23–25]. This study provides new observations regarding duration of antidiabetic drugs, including metformin, insulin and SU. The results demonstrate a persistently decreased risk of PCa for all anti-diabetic medications and durations. One possible explanation for this observation is that metformin and insulin are thought to reduce the activity of mTOR, which blocks progression of the cell cycle and cancer growth [26, 27]. For example, Pircher et al. conducted a study exploring antidiabetic drugs that influence molecular mechanisms in prostate cancer and showed that insulin use significantly decreased active mTOR in cancer tissue [26]. In addition, they found an AMPK and AKT independent regulation of the mTOR pathway, as there were no differences in pAKT levels among treatment groups (insulin and metformin groups) [26]. Hence, medication that can attenuate mTOR activity with subsequent blockade of cell cycle progression, can potentially also block the progression of the prostate cell cycle and cancer growth [28]. However, some observational studies have shown no protective effect of metformin and insulin on PCa risk [14, 29–31]. These studies did not take selection bias and confounding into account. Studies examining the impact of SU on PCa risk have provided inconsistent results [11, 32, 33]. No previous study has examined SU separately from other antidiabetic drugs or examined the impact of its duration on PCa risk, as we have here.

### Risk categories of PCa

We found a consistently stronger inverse association of duration of T2DM and antidiabetic medications with risk of low and intermediate-risk PCa. This association was not observed for high-risk PCa, regional and distant metastatic PCa. These results are consistent with previous large population-based studies [23, 33, 34], which concluded that neither T2DM nor antidiabetic medications were associated with a reduction in risk of aggressive PCa. Conversely, some studies [35–38] have found that T2DM is associated with increased risk of high-grade PCa. The biological mechanism behind this remains unclear [14].

It is essential to consider detection bias when evaluating the association of T2DM and antidiabetic treatments with different risk categories of PCa. Early detection of PCa depends on PSA testing and subsequent biopsy of the prostate. PSA levels have been shown to be lower in T2DM patients than in non-diabetic men [39]. Therefore, PSA is less sensitive to detect early-stage PCa in men with T2DM. Prostate size among PCa men with T2DM has been reported to be larger than in men without T2DM, which may affect biopsy results [40]. A larger prostate may dilute the cancer cells, which may cause a decrease in the probability of the detection of small indolent cancers in the low-risk category group using biopsy [39]. These two observations may affect PCa detection at an early stage, which may partially explain the decreased risk of low-risk PCa among men with T2DM. Men with T2DM who developed into PCa are therefore more likely to be diagnosed at a more advanced stage.

### Strengths/limitations

The strength of this study is the use of data from a national-wide cohort in Sweden, which has been linked to other national high-quality registers and demographic databases including the Longitudinal integrated database for health insurance and labour market studies, the National Diabetes Register and the National Prescribed Drug Register. Since previous PCBaSe studies were performed, there has been a substantial improvement in the completeness of the National Diabetes Register [41], which make the data in this study more representative. We also included information from the Prescribed Drug Registry, which comprises all out-patients filled prescription data since July 2005 [41]. We were also able to investigate the association of different anti-diabetic medications including high-dose and low-dose metformin with PCa risk using detailed prescription data from the National Prescribed Drug Register. Thirdly, our study had a longer follow-up (8.5 years) than other studies where median follow-up was 5–7 years [14, 29, 42]. A limitation of our study is that there was insufficient power to investigate associations of exposure times to antidiabetic medications with low-risk/ intermediate-risk/ high-risk PCa given the smaller number of men with PCa in these specific risk categories. Secondly, our study may be prone to residual confounding (e.g., family history of T2DM and PCa), although we obtained detailed information of participants’ socio-economic status from the Longitudinal integrated database for health insurance and labour market studies database. Thirdly, approximately 6% of participants had missing data on the date of T2DM diagnosis and registration in the National Diabetes Register registry. We used duration of antidiabetic medications in the National Prescribed Drug Register to replace these missing values, which may result in a shorter interval between T2DM diagnosis and PCa diagnosis.

## Conclusion

There was a persistent decreased risk of PCa across all anti-diabetic medications studied and across all durations of T2DM. This association was strongest for risk of low- and intermediate risk PCa and not observed for high-risk PCa. Given the prevalence of T2DM worldwide, it is important to note that T2DM and its medication may protect some men from PCa, though not necessarily from developing high risk disease. However, the interplay between T2DM and PCa warrants further study.

## Data Availability

The data that support the findings of this study are available from PCBaSe Sweden, but restrictions apply to the availability of these data, which were used under license for the current study, and so are not publicly available. Data are however available from the authors upon reasonable request and with permission of PCBaSe Sweden.

## Abbreviations

PCa: Prostate cancer
PSA: Prostate-Specific Antigen
T2DM: Type 2 diabetes mellitus
SU: Sulfonylurea
CCI: Charlson Comorbidity Index
OR: Odds ratio
95% CI: 95% confidence interval
PCBaSe: Prostate Cancer data Base Sweden
